# Community gut colonization by *tet*(X4)-positive multidrug-resistant *Escherichia coli* in healthy individuals from urban residents in Shenzhen, China

**DOI:** 10.3389/fcimb.2025.1667196

**Published:** 2025-10-07

**Authors:** Ruoyan Peng, Pei Liang, Wenxiao Jiang, Zhaodong Li, Li Wang, Yi Huang, Xianfang Zhang, Yijia Guo, Ying Wang, Jing Wang, Jiubiao Guo, Feifei Yin, Dachuan Lin

**Affiliations:** ^1^ Hainan Medical University–The University of Hong Kong Joint Laboratory of Tropical Infectious Diseases & Key Laboratory of Tropical Translational Medicine of Ministry of Education, College of Basic Medical Sciences, Hainan Medical University, Haikou, China; ^2^ Department of Nutrition and Food Hygiene, School of Public Health, Shenzhen University, Shenzhen, China; ^3^ Institute of Clinical Medicine, The Second Affiliated Hospital of Hainan Medical University, Haikou, China; ^4^ The University of Hong Kong, Shenzhen, China; ^5^ School of Public Health, Xinjiang Medical University, Urumqi, China; ^6^ Clinical Research Center, The First Affiliated Hospital of Shantou University Medical College, Shantou, China

**Keywords:** community, healthy carriers, tigecycline resistance, *E. coli*, plasmids

## Abstract

**Background:**

Tigecycline remains a last-resort antibiotic for treating multidrug-resistant (MDR) Gram-negative pathogens. The emergence of *tet*(X4)-mediated high-level tigecycline resistance in *Escherichia coli* has raised global concern, yet its prevalence in healthy human populations remains limited.

**Methods:**

We conducted a community-based surveillance study involving 245 fecal samples from healthy individuals in three urban communities in Shenzhen, China. Tigecycine-resistant strains were isolated using MacConkey agar supplemented with 2 mg/L tigecycline and confirmed by PCR detection of *tet*(X). Antimicrobial susceptibility testing, whole-genome sequencing (WGS), and phylogenetic analysis were performed.

**Results:**

Tigecycline-resistant *E. coli* were detected in 1.6% (4/245) of samples. All isolates carried *tet*(X4) and exhibited an MDR phenotype. WGS revealed that *tet*(X4) was located on IncY (n=1) and IncFIA8-IncHI1/ST17 plasmids (n=3), which closely resembled previously described plasmids and co-harbored additional resistance genes. The core *tet*(X4)-carrying region in all four plasmids, associated with IS*CR2*, was highly similar to that of p47EC—the first *tet*(X4)-bearing plasmid identified in porcine *E. coli* in China. Notably, the three IncFIA-IncHI1/ST17 plasmids shared an identical 12,536-bp region structured as IS*1*–*catD*–*tet*(X4)–IS*CR2*–ΔIS*CR2*–*floR*–ΔIS*CR2.* Virulence-associated genes involved in adhesion, iron acquisition, biofilm formation, and secretion systems were also identified in four *tet*(X4)-positive isolates. The four isolates belonged to globally distributed sequence types ST10, ST201, ST877, and ST1308. Phylogenomic analysis demonstrated close genetic relatedness between these community isolates and strains from diverse geographical regions and hosts.

**Conclusions:**

This study reveals silent intestinal colonization by *tet*(X4)-positive MDR *E. coli* among healthy urban residents, highlighting the role of community reservoirs in the dissemination of last-resort antibiotic resistance. These findings underscore the urgent need for One Health-oriented antimicrobial resistance surveillance and intervention strategies that extend beyond clinical settings.

## Introduction

Tigecycline, a 9-t-butylglycylamido derivative of minocycline, is a glycylcycline antibiotic that inhibits bacterial protein synthesis by binding to the 30S ribosomal subunit (Yaghoubi et al., 2021). This mechanism allows tigecycline to evade traditional tetracycline resistance determinants, making it as a last-resort therapeutic agent for infections caused by multidrug-resistant (MDR) pathogens (Yaghoubi et al., 2021). However, the increasing emergence of tigecycline resistance is compromising its clinical utility.

Of particular concern is the *tet*(X) family of flavin-dependent monooxygenase genes, which inactivate tigecycline through enzymatic degradation (Aminov, 2021). Among them, the plasmid-mediated *tet*(X4), first identified in *Escherichia coli* from swine, confers high-level tigecycline resistance and facilitates horizontal gene transfer across diverse bacterial hosts and ecological inches (He et al., 2019). The widespread detection of *tet*(X4) in both clinical and agricultural settings, particularly among *E. coli* strains, underscores its growing public health threat (Li et al., 2023).

Although *tet*(X4)-positive *E. coli* has been increasingly reported in food-producing animals—a trend linked to the historical and extensive use of tetracyclines in agriculture—as well as in food products, and human patients (Aminov, 2021; Li et al., 2023), data on its carriage in healthy human populations, especially in community settings, remain limited (Ding et al., 2020, 2024; Dong et al., 2021). The human gut microbiota serves as a significant but underexplored reservoir for antimicrobial resistance genes (ARGs) (Carlet, 2012; Donskey, 2004). Within this niche, horizontal gene transfer can facilitate the dissemination of resistance determinants across environmental, zoonotic, and clinical bacterial populations (McInnes et al., 2020). While antimicrobial misuse and overuse are known drivers of resistance emergence (Allcock et al., 2017; Ferrara et al., 2024), it remains unclear whether AGRs, such as *tet*(X4), can persist or evolve in community-dwelling individuals without direct antibiotic exposure. This uncertainty is particularly relevant given that tetracyclines are poorly metabolized and can persist in the environment, potentially exerting low-level selective pressure through dietary or environmental exposure, even in the absence of clinical antibiotic use (Allcock et al., 2017).

Given the potential role of asymptomatic carriers in the silent spread of tigecycline resistance, enhanced surveillance of *tet*(X4) in healthy populations is urgently needed. In this study, we investigated tigecycline-resistant *E. coli* isolated from fecal samples of 245 asymptomatic adults residing in three urban communities in Shenzhen, China. Our objectives were to assess the prevalence of tigecycline resistance, characterize the genetic and plasmid features of *tet*(X4)-positive strains, and explore potential epidemiological links to clinical and agricultural sources. These findings offer important insight into the community-level dissemination of tigecycline resistance and highlight the need to broaden antimicrobial resistance (AMR) surveillance beyond clinical settings.

## Materials and methods

### Bacterial isolation and detection of the *tet*(X) gene

In October 2022, 245 fecal samples were obtained from healthy individuals aged 16 to 79 years, who had no self-reported symptoms of acute infection (e.g., diarrhea, fever, respiratory or urinary tract infections), no history of hospitalization or surgery in the past three months, and no antibiotic use within the preceding three months, across three residential communities in Shenzhen to investigate the prevalence of tigecycline-resistant *Enterobacteriaceae* (**Supplementary Table S1**). Samples were directly inoculated into LB broth and incubated at 37°C for 12–18 h for enrichment. Enriched cultures were then streaked onto LB agar plates containing 2 μg/mL tigecycline and incubated at 37°C for 12–18 h. Presumptive colonies were purified by subculturing, and bacterial species were identified using the VITEK-2 automated microbial identification system (bioMérieux, Lyon, France). The presence of the *tet*(X) gene was screened by PCR and Sanger sequencing using universal primers *tet*(X)-F (5′-CCGTTGGACTGACTATGGC-3′) and *tet*(X)-R (5′- TCAACTTGCGTGTCGGTAA-3′), as previously described (Wang et al., 2019).

### Antimicrobial susceptibility testing

Antimicrobial susceptibility profiles were determined using the broth microdilution or the agar dilution method according to the guidelines of the Clinical and Laboratory Standards Institute (CLSI). Minimum inhibitory concentrations (MICs) were assessed for 14 antibiotics: ampicillin, cefotaxime, meropenem, gentamicin, amikacin, streptomycin, tetracycline, tigecycline, chloramphenicol, nalidixic acid, ciprofloxacin, colistin, fosfomycin, and sulfamethoxazole-trimethoprim. MIC breakpoints for streptomycin and tigecycline were interpreted based on the European Committee on Antimicrobial Susceptibility Testing (EUCAST) criteria (https://www.eucast.org/), while those for other agents followed the 33^rd^ edition of the CLSI document M100 (CLSI, 2023). *E. coli* ATCC 25922 was used as the quality control strain.

### Conjugation assay

To assess the horizontal transferability of the *tet*(X4) gene, conjugation experiments were performed using *tet*(X)-positive isolates as donor strains and a high-level streptomycin-resistant *E. coli* strain C600 as the recipient. Briefly, donor and recipient strains were separately cultured in 2 mL LB broth at 37 with shaking (180 rpm) for 4 h, mixed at a 1:4 (v/v) ratio and incubated statically for 24 h. The cultures were then centrifuged at 5,000 × g for 5 min, the supernatant discarded, and the pellet resuspended in sterile PBS. Appropriate dilutions (100 μL) were plated on selective agar containing tigecycline (2 mg/mL) and streptomycin (3000 mg/mL) to select for transconjugants. Colonies were incubated at 37°C for 16–24 h and confirmed as transconjugants by PCR detection of the *tet*(X) gene as described above. All experiments were performed in triplicate.

### Whole-genome sequencing and bioinformatics analysis

The same DNA extraction protocol was applied to both Illumina and Nanopore sequencing to ensure data consistency and comparability. Genomic DNA was extracted from *E. coli* isolates using the PureLink Genomic DNA Mini Kit (Invitrogen, USA). Short-read sequencing libraries were prepared using the Illumina NovoSeq PE150 platform (2×150 bp paired-end), while long-read sequencing was performed using the Oxford Nanopore MinION platform (Oxford Nanopore Technologies, UK). Hybrid genome assemblies were generated using Unicycler (v 0.5.0) (Wick et al., 2017) and subsequently corrected with Pilon (v 1.24) (Walker et al., 2014). Plasmid replicon types were identified using PlasmidFinder (Carattoli et al., 2014), and antibiotic resistance genes, including chromosomal mutations mediating resistance, were annotated using ResFinder (Bortolaia et al., 2020) and PointFinder (Zankari et al., 2017). Multilocus sequence typing (MLST) were assigned via MLST analysis (Larsen et al., 2012). Virulence factors were identified using ABRicate v0.8 with the VFDB database (updated October 2020). Comparative analysis of *tet*(X)-carrying plasmids and related plasmids was performed and visualized using BRIG (Alikhan et al., 2011).

A phylogenetic tree based on core genome single nucleotide polymorphism (cgSNP) was constructed using Parsnp v1.5.4 (https://github.com/marbl/parsnp) and visualized with iTOL (https://itol.embl.de/index.shtml). Our *tet*(X4)-positive *E. coli* isolate served as the reference genome for phylogenetic tree construction. To identify relevant strains, we retrieved *E. coli* isolates of the same ST from the NCBI database. cgSNP distances between the isolates sharing the same ST were calculated using Snippy v4.6.0 (https://github.com/tseemann/snippy), and only those with ≤200 SNPs relative to the reference were included in the final phylogenetic analysis.

### Nucleotide sequence accession number

The whole-genome sequences of the four *tet*(X4)-positive isolates have been deposited in GenBank under accession number: PRJNA1288486.

## Results

### Prevalence of tigecycline-resistant *E. coli* isolates in healthy individuals

Tigecycline-resistant Enterobacteriaceae were identified in 4 out of 245 fecal samples collected from healthy individuals, yielding a prevalence rate of 1.63%. All isolates were confirmed as *E. coli* by both the VITEK2 automated identification system. PCR amplification and Sanger sequencing verified the presence of the *tet*(X4) gene in all four isolates. Notably, three isolates (SZ22HTE1, SZ22HTE2, and SZ22HTE3) were recovered from individuals residing in the same community (Dawang), whereas the fourth isolate (SZ22HTE4) originated from a separate community (Fenghua).

### Phenotypic and genotypic characterization of antimicrobial resistance

All four *E. coli* isolates exhibited resistance to tigecycline, with MICs ranging from 8 to 32 mg/L (**Supplementary Table S2**). They also displayed resistance to multiple antibiotics, including ampicillin, streptomycin, tetracycline, and chloramphenicol (**Table 1**). Additionally, three isolates (SZ22HTE1–SZ22HTE3) were resistant to sulfamethoxazole/trimethoprim, whereas SZ22HTE4 remained susceptible. In contrast, all isolates were susceptible to cefotaxime, meropenem, gentamicin, amikacin, nalidixic acid, ciprofloxacin, colistin, and fosfomycin (**Supplementary Table S2**). According to the standard definition—resistance to at least one agent in three or more antimicrobial classes—all isolates were classified as multidrug-resistant (MDR).

**Table 1:** Characterization of tigecycline-resistant *Escherichia coli* isolates in this study.

Strain	ST	Resistance patterns	Resistance genes	Location of tet(X4)
SZ22HTE1	201	AMP/STR/TET/TIL/CHL/SXT	*bla*_TEM-1B_, *aadA1*, *aadA2*, *tet*(X4), *tet*(A), *tet*(M), *cmlA1*, *floR*, *qnrS1*, *sul2*, *sul3*, *dfrA12*, *erm*(42)	pSZ22HTE1-1 (IncY, 106,177 bp)
SZ22HTE2	877	AMP/STR/TET/TIL/CHL/SXT	*bla*_TEM-1B_, *aadA1*, *aadA2*, *aadA22*, *strA*, *strB*, *tet*(X4), *tet*(B), *cmlA1*, *floR*, *qnrS1*, *sul3*, *dfrA12*, *lnu*(G)	pSZ22HTE2-1 (IncFIA8-IncHI1/ST17, 190,711 bp)
SZ22HTE3	10	AMP/STR/TET/TIL/CHL/SXT	*bla*_TEM-1B_, *aadA2*, *aadA22*, *tet*(X4), *tet*(A), *floR*, *qnrS1*, *sul3*, *dfrA12*, *lnu*(G)	pSZ22HTE3-1 (IncFIA8-IncHI1/ST17, 201,009 bp)
SZ22HTE4	1308	AMP/STR/TET/TIL/CHL	*bla*_TEM-1B_, *aadA22*, *tet*(X4), *tet*(A), *tet*(M), *floR*, *qnrS1*, *lnu*(G), *erm*(42)	pSZ22HTE4-1 (IncFIA8-IncHI1/ST17, 192,057 bp)

AMP, Ampicillin; STR, Streptomycin; TET, Tetracycline; TIL, Tigecycline; CHL, Chloramphenicol; SXT, Trimethoprim-sulfamethoxazole.

WGS identified resistance genes that largely correlated with phenotypic profiles (**Table 1**). The presence of *tet*(X4), previously confirmed by PCR and Sanger sequencing, was further validated by WGS, explaining the observed tigecycline resistance. Additional tetracycline resistance genes, including *tet*(A), *tet*(B), and *tet*(M), were variably present and matched the tetracycline-resistant phenotypes. All isolates carried *bla*_TEM-1B_, consistent with ampicillin resistance, and aminoglycoside resistance genes (*aadA1*, *aadA2*, *aadA22*, and *strA*/*strB*) aligned with streptomycin resistance. Resistance to chloramphenicol was associated with *cmlA1* and/or *floR*.

The plasmid-mediated quinolone resistance gene *qnrS1* was identified in all isolates, but no chromosomal mutations were found within the quinolone resistance-determining region, consistent with their susceptibility to fluoroquinolones. Sulfonamide resistance genes (*sul2*, *sul3*) and the trimethoprim resistance gene *dfrA12* were found only in SZ22HTE1–SZ22HTE3, in agreement with their resistance to sulfamethoxazole/trimethoprim. Additionally, all isolates harbored macrolide-lincosamide resistance genes *lnu*(G) and/or *erm*(42).

### Virulence gene profiles of *tet*(X4)-positive *E. coli* isolates

All four *E. coli* isolates harbored multiple virulence-associated genes involved in adhesion, iron acquisition, biofilm formation, motility, and secretion systems, which are critical for bacterial colonization and pathogenicity (**Table 2**). Adhesion genes such as *fimH*, *ehaB*, *upaG*/*ehaG*, *csgG*, and *cgsF* were variably distributed among the isolates, supporting host cell attachment. Iron acquisition genes, including *entE*, *fepD*, *entC*, *fepG*, *fepC*, and *entB*, were also detected in a strain-specific manner, enabling iron scavenging essential for survival in host environments. The toxin gene *hlyE*/*clyA*, identified only in SZ22HTE1, may contribute to host cell lysis and tissue damage.

**Table 2:** Virulence genes of tigecycline-resistant *Escherichia coli* isolates in this study.

Strain	Adhesion	Iron Acquisition	Toxins	Biofilm	Motility	Secretion System
SZ22HTE1	*fimH*	*entE*, *fepD*	*hlyE*/*clyA*	*agn43*, *cah*	*flhA*, *ta*	*aec27*/*clpV*, *aec15*, *tssM*
SZ22HTE2	*ehaB*, *upaG*/*ehaG*	*entC*, *fepG*, *fepC*	–	*cgsG*	*flhB*	*clpV*, *espX4*
SZ22HTE3	*upaG*/*ehaG*, *csgG*, *cgsF*	*entB*	–	*cgsG*	*fliA*	*clpV*, *espX1*
SZ22HTE4	*upaG*/*ehaG*	*entE*, *entC*	–	–	–	*aec27*/*clpV*, *tssM*, *espX5*

Gene associated with biofilm formation (*agn43*, *cah*, and *cgsG*) were detected in SZ22HTE1 to SZ22HTE3 and may enhance persistence and immune evasion. Motility-related genes (*flhA*, *flhB*, and *fliA*) were present in the same three isolates, likely facilitating bacterial movement and invasion. Secretion system genes (*aec27/clpV*, *aec15*, *tssM*, *espX4*, *espX1*, and *espX5*) were identified in various combinations across all four isolates and may facilitate the delivery of virulence factors into host cells (**Table 2**). Although the specific virulence gene profiles varied among isolates, each strain possessed multiple functional categories of virulence factors, underscoring their potential to cause clinically relevant infections.

### Characterization of the *tet*(X4)-carrying plasmid in *E. coli* strains

**Figure 1:**
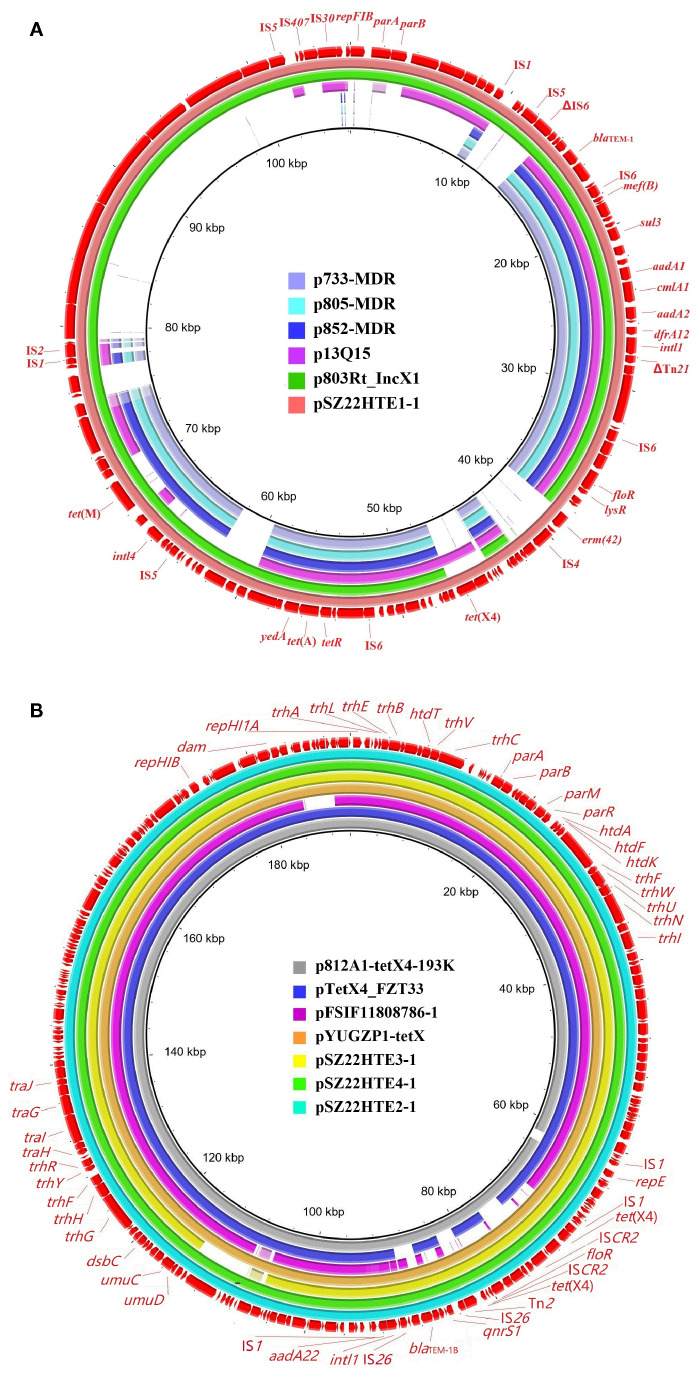
Sequence comparison of *tet*(X4)-bearing plasmids in this study with other similar plasmids using BRIG. **(A)** IncY plasmid pSZ22HTE1-1; **(B)** IncFIA8-IncHI1/ST17 plasmids pSZ22HTE2-1, pSZ22HTE3-1, and pSZ22HTE4-1. The outer circles in red with annotation are the reference plasmids pSZ22HTE1–1 and pSZ22HTE2-1, respectively.

All four isolates carried multiple plasmids with diverse replicon types and antimicrobial resistance genes (**Supplementary Table S3**). In strain SZ22HTE1, the *tet*(X4) gene was located on the largest plasmid, pSZ22HT1-1 (IncY, 106,177 bp), which also harbored 11 additional resistance genes *bla*_TEM-1B_, *aadA1*, *aadA2*, *tet*(A), *tet*(M), *cmlA1*, *floR*, *sul2*, *sul3*, *dfrA12*, and *erm*(42). The *tet*(X4)-carrying IncY plasmid pSZ22HT1–1 exhibited high sequence similarity (>99.9%) to plasmid p803Rt_IncX1 (CP080067) isolated from a human-derived *E. coli* strain in Shenzhen, China with 94% coverage, and plasmid p13Q15 (ON934549) from an *E. coli* strain in Guangdong, China with 53% coverage (**Figure 1A**).

The remaining three isolates (SZ22HTE2, SZ22HTE3, and SZ22HTE4) each carried a *tet*(X4)-positive hybrid IncFIA8-IncHI1/ST17 plasmid, designated pSZ22HT2-1 (190,711 bp), pSZ22HT3-1 (201,009 bp), and pSZ22HT4-1 (192,057 bp), respectively. These plasmids also carried resistance genes *bla*_TEM-1B_, *aadA22*, *qnrS1*, and *floR*. Comparative genomic analysis revealed that the *tet*(X4)-bearing IncFIA8-IncHI1/ST17 plasmids from SZ22HTE2, SZ22HTE3, and SZ22HTE4 were closely related to multiple plasmids, including *tet*(X4)-carrying plasmids p812A1-tetX4-193K (CP116047, *E. coli*, China), pYUGZP1-tetX (pig, *E. coli*, China) from pig source, and pTetX4_FZT33 (CP132725, *E. coli*, China) from hospital sewage (**Figure 1B**).

However, conjugation assays using *E. coli* C600 as the recipient strain failed to produce transconjugants under the tested conditions, indicating that these *tet*(X4)-bearing plasmids were either non-conjugative or required specific conditions or helper plasmids for mobilization.

### Variation in the genetic environment of *tet*(X4)

**Figure 2:**
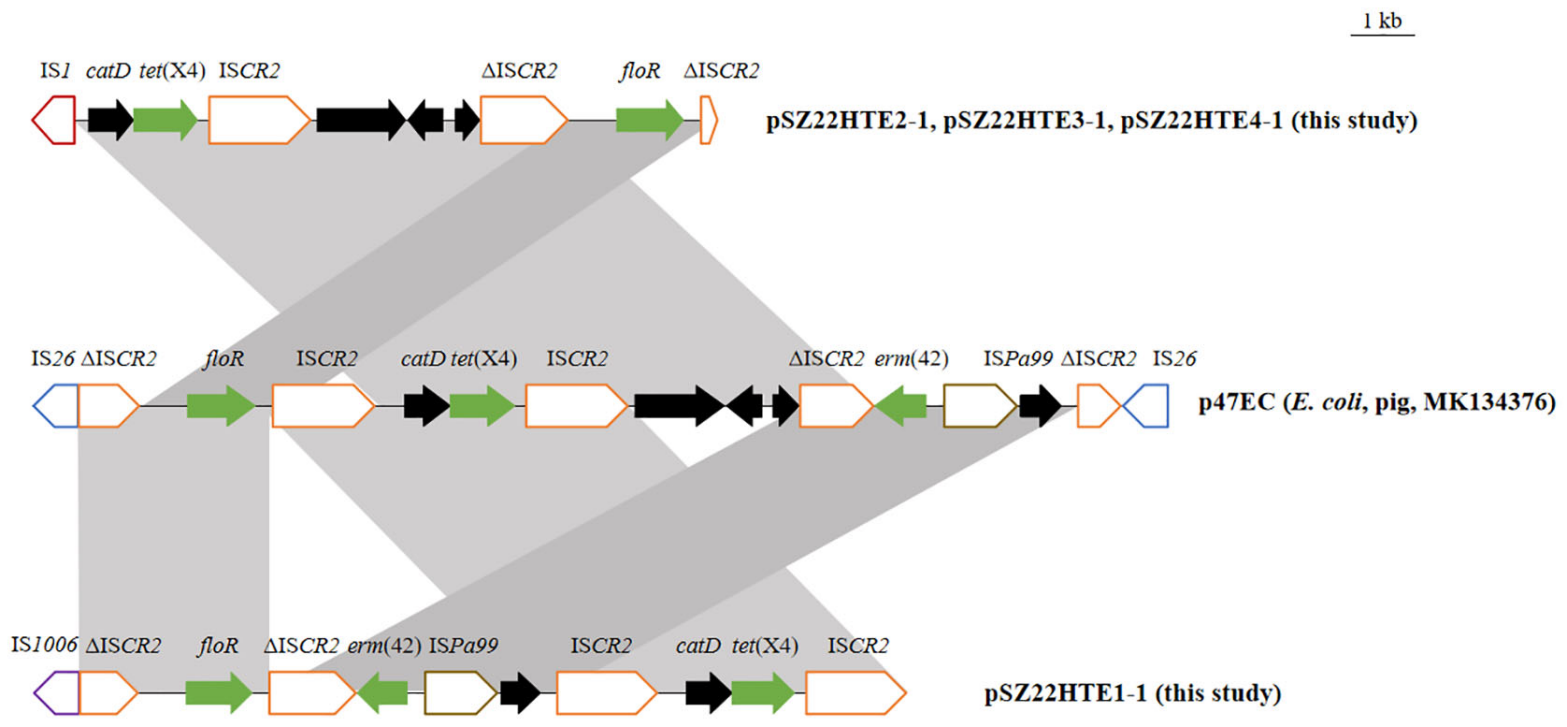
Genetic structures of the *tet*(X4) gene in this study and comparison with p47EC (MK134376). The extent and directions of genes are indicated by arrows. Antibiotic resistance genes are shown in red. Truncated mobile elements are marked with a “Δ” symbol. Insertion sequences (ISs) are represented as boxes labeled with their names. Regions with >99% identity are shaded in gray.

As shown in **Figure 2**, the *tet*(X4) gene in the three IncFIA-IncHI1/ST17 plasmids (pSZ22HT2-1, pSZ22HT3-1, and pSZ22HT4-1) was embedded in an identical 12,536-bp region organized as IS*1*–*catD*–*tet*(X4)–IS*CR2*–ΔIS*CR2*–*floR*–ΔIS*CR2.* While the core *tet*(X4)-containing structure closely resembled that of p47EC, the first reported *tet*(X4)-bearing plasmid isolated from *E. coli* of porcine origin in China (He et al., 2019), several notable differences were observed. Most prominently, IS*CR2* replaced the upstream IS*1* element in p47EC. Furthermore, the chloramphenicol resistance gene *floR*, associated with an incomplete IS*CR2*, was located downstream of the *tet*(X4) structure in our plasmids, in contrast to its upstream position in p47EC.

Plasmid pSZ22HTE1–1 exhibited a different but related arrangement. It retained the conserved IS*CR2*–*catD*–*tet*(X4)–IS*CR2* structure found in p47EC, but its upstream region contained an additional resistance module carrying both *floR* and *erm*(42). In comparison, p47EC carried the ΔIS*CR2-erm*(42)-IS*Pa99* segment upstream of the *tet*(X4) conserved segment, but in the opposite orientation. These structural variations underscore the genetic plasticity of *tet*(X4)-associated regions and their potential for mobilization and dissemination across diverse plasmid backgrounds driven by mobile genetic elements.

### Phylogenomic analysis of *tet*(X4)-positive *E. coli* strains

**Figure 3:**
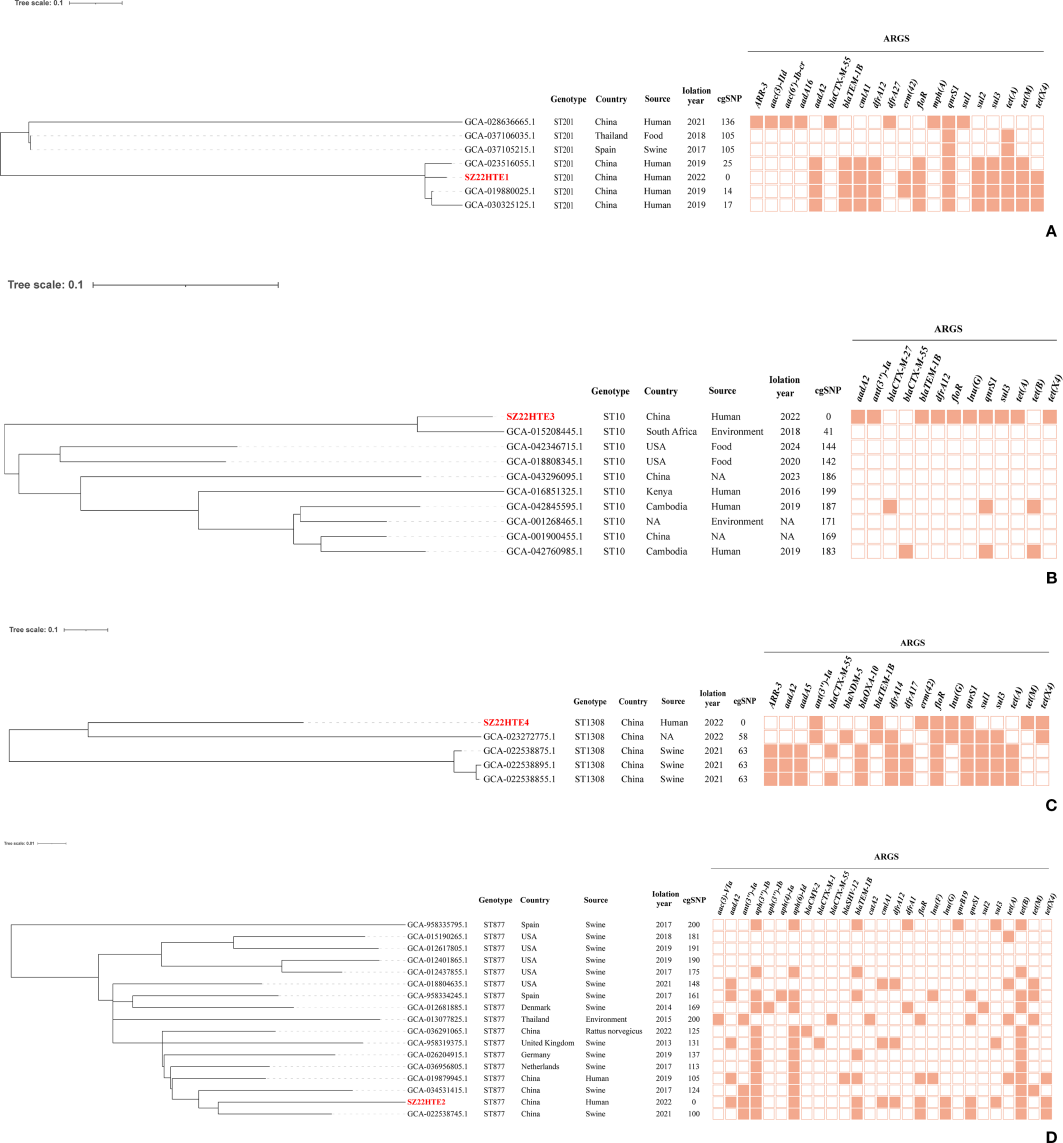
Core-genome SNP phylogeny of four *tet*(X4)-positive *E*. *coli* isolates (in red) with the same ST strains with ≤200 SNPs. **(A)** ST201; **(B)** ST10; **(C)** ST1308; **(D)** ST877. Branch lengths indicate SNP distances; key ARGs are annotated.

Four *tet*(X4)-positive *E. coli* isolates in this study were assigned to four sequence types (STs): ST10, ST201, ST877, and ST1308. To investigate the genetic relatedness between these *E. coli* isolates and publicly available *E. coli* strains of the same ST, we conducted a phylogenomic analysis based on cgSNPs. The resulting phylogeny revealed that our community-derived isolates were closely related to *E. coli* strains from diverse geographical regions and hosts (**Figure 3**).

Our *tet*(X4)-positive ST201 isolate (SZ22HTE1) exhibited high genetic similarity to three human clinical ST201 isolates from China (GCA_023516055.1, GCA_019880025.1, and GCA_030325125.1), differing by only 14–25 SNPs and carrying identical or similar antimicrobial resistance genes, suggesting a potential epidemiological link (**Figure 3A**). SZ22HTE1 also showed relatively limited divergence (105 and 136 SNPs) from *E. coli* strains of porcine (GCA_037105215.1, Spain), food (GCA_037106035.1, Thailand), and clinical (GCA_028636665.1, China) origin (**Figure 3A**), further indicating potential inter-host and inter-regional transmission.

Our ST10 *tet*(X4)-positive isolate, SZ22HTE3, clustered with nine ST10 *E. coli* isolates from diverse countries and sources. It was most closely related (41 SNPs) to an environmental isolate from South Africa. In contrast, it differed from the remaining eight isolates by 142–199 SNPs, suggesting a certain level of genomic divergence among ST10 isolates (**Figure 3B**). For ST1308, five isolates, including our community-derived strain SZ22HTE4, formed a tight phylogenetic cluster with minimal genetic SNP differences of only 58 or 63, suggesting recent common ancestry or possible transmission events. Among the four closely related isolates, three were isolated from swine in China (GCA_022538875.1, GCA_022538895.1, GCA_022538855.1), and one had an unreported source (GCA_023272775.1) (**Figure 3C**). By comparison, the ST877 lineage exhibited greater genetic heterogeneity. Compared with our *tet*(X4)-positive ST877 isolate (SZ22HTE2), the other 16 ST877 *E. coli* isolates displayed broader genomic divergence, with SNP differences ranging from 100 to 200, highlighting higher diversity within this ST (**Figure 3D**).

## Discussion

Since its approval by the U.S. Food and Drug Administration in 2005, tigecycline has served a last-resort antibiotic for the treatment of severe infections caused by MDR bacteria, particularly carbapenem-resistant *Enterobacteriaceae* (Yaghoubi et al., 2021). However, the growing prevalence of tigecycline resistance has become a significant clinical and public health concern. Among the known resistance mechanisms, the plasmid-mediated *tet*(X4) gene has gained particular attention due to its ability to confer high-level tigecycline resistance and its rapid dissemination across bacterial species and ecological niches through horizontal gene transfer (Dong et al., 2021; He et al., 2019; Li et al., 2023).

Globally, *tet*(X4)-carrying *E. coli* strains have been detected in clinical, animal, food, and environmental settings (Li et al., 2023). However, their presence in healthy human populations, particularly in urban communities, remains insufficiently characterized. Our study identified a *tet*(X4)-positive *E. coli* colonization rate of 1.6% (4/245) in fecal samples from healthy individuals in Shenzhen, a densely populated metropolitan area. Although this prevalence is considerably lower than that observed in animal-derived (18.24%) and food-derived (20.6%) *E. coli* isolates, it exceeds the rates reported in clinical patients (0.07–0.1%) (Bai et al., 2019; He et al., 2019; Li et al., 2021), suggesting that the human gut may serve as a previously underappreciated reservoir for plasmid-mediated tigecycline resistance. These findings highlight the necessity of enhanced AMR surveillance in non-clinical community populations to detect early signs of resistance dissemination.

AMR is an escalating global health crisis, contributing to an estimated 30,000 deaths annually in the EU and nearly 5 million worldwide in 2019 (Cassini et al., 2019; Ho et al., 2025). The human gastrointestinal tract, comprising a dense and diverse microbial community (~10¹^4^ cells across approximately 1, 000 bacterial species), is a hotspot for the acquisition, persistence, and horizontal transfer of ARGs (Despotovic et al., 2023; Lynch and Pedersen, 2016). The gut microbiome not only reflects the local AMR burden but also influences patient outcomes, particularly in critically ill individuals with gut barrier dysfunction or acute gastrointestinal injury, where microbiome disruption and elevated ARG levels are associated with worse clinical outcomes (Bai et al., 2025). Notably, *E. coli*, a core member of the intestinal microbiota and a widely used sentinel organism in AMR surveillance, readily acquires ARGs from food, animal, and environmental sources, and can transfer them to pathogenic bacteria such as *Shigella* and *Klebsiella* through plasmids or other mobile genetic elements (Thanh Duy et al., 2020). In our study, three isolates (SZ22HTE2, SZ22HTE3, and SZ22HTE4) harbored the epidemic IncFIA8-IncHI1/ST17 plasmid carrying *tet*(X4), suggesting early-stage horizontal dissemination of tigecycline resistance within the community gut microbiota of an urban community. These plasmids co-harbored additional resistance genes, resulting in MDR phenotypes and enabling co-selection and persistence in diverse hosts (Lu et al., 2018; Yan et al., 2024). Notably, the epidemic IncFIA8-IncHI1/ST17 plasmid identified in this study shows high homology to pRDZ41 (CP139495.1) from *Klebsiella pneumoniae*, providing direct evidence for its potential to disseminate *tet*(X4) into this high-risk pathogen.

Importantly, these *E. coli* isolates possessed a range of virulence-associated genes related to adhesion (e.g., *fimH*, *ehaB*, *upaG*/*ehaG*), iron acquisition (e.g., *entE*, *fepD*), biofilm formation (e.g., *agn43*, *cah*), motility (e.g., *flhA*, *fliA*), and secretion systems (e.g., *aec27*/*clpV*, *tssM*). The coexistence of virulence factors and resistance determinants in community-derived *E. coli* is particularly alarming, as it increases the risk of difficult-to-treat infections and facilitates ARG dissemination into high-risk clinical pathogens.

Although the healthy human gut microbiota may provide colonization resistance against invasive AMR bacteria—potentially via mechanisms such as microbiome-mediated nutrient depletion (Isaac et al., 2022; Le Guern et al., 2021)—our findings underscore the vulnerability of even healthy individuals to colonization by plasmid-mediated tigecycline-resistant *E. coli* strains. This silent carriage may act as a hidden conduit for ARG dissemination between community and healthcare environments, presenting significant challenges for infection prevention and control.

WGS further revealed that our *tet*(X4)-positive isolates belonged to globally prevalent *E. coli* STs, e.g., ST10, ST877, and ST1308, frequently associated with MDR phenotypes and detected in both human and animal hosts (Elias et al., 2019; García et al., 2018; Liu et al., 2024). The detection of these epidemic STs in healthy individuals, along with their close genetic relatedness to isolates from clinical, food, animal, and the environmental sources, reinforces a One Health perspective. Phylogenomic analysis demonstrated close genetic relatedness between our community isolates and strains from diverse geographic regions and hosts (e.g., swine, food, environment), reinforcing the role of cross-sectoral transmission in the spread of *tet*(X4). This underscores the interconnectedness of AMR reservoirs across ecosystems and highlights the urgent need for integrated, cross-sectoral genomic surveillance.

Despite the valuable insights provided, this study has several limitations. Only three urban communities were sampled, and the number of *tet*(X4)-positive isolates was relatively small, limiting the generalizability of our findings. Additionally, although the plasmid structures were well-characterized, functional studies such as assessing plasmid transfer under different conditions or colonization capacity *in vivo* were not performed. The observed lack of successful conjugation *in vitro* highlights the need for further investigation into the mobility mechanisms of *tet*(X4)-bearing plasmids. Further studies should explore whether these plasmids can successfully conjugate by employing diverse recipient strains, co-introducing helper plasmids, or optimizing mating conditions (e.g., modifying incubation temperature, adjusting donor-to-recipient ratios, or using filter mating assays) under alternative experimental settings. Importantly, future investigations should include larger-scale, longitudinal surveillance, and mechanistic assessments to better understand the dynamics and risks of *tet*(X4)-positive *E. coli* colonization in healthy populations.

## Conclusion

In summary, our findings highlight the emergence of *tet*(X4)-positive MDR *E. coli* in the intestinal microbiota of healthy individuals from urban communities. These isolates co-harbored multiple ARGs and virulence determinants, highlighting their potential to cause difficult-to-treat infections and to act as reservoirs for further resistance dissemination. The detection of globally circulating high-risk clones such as ST10 and ST201 in asymptomatic carriers underscores the silent spread of tigecycline resistance in non-clinical settings. These findings call for urgent and coordinated surveillance strategies beyond hospital environments, in alignment with One Health principles, to contain the spread of last-resort antibiotic resistance and safeguard public health.

## Data Availability

The datasets presented in this study can be found in online repositories. The names of the repository/repositories and accession number(s) can be found in the article/**Supplementary Material**.
