# Supplementary material for: Community gut colonization by tet(X4)-positive multidrug-resistant Escherichia coli in healthy individuals from urban residents in Shenzhen, China
Source: Front Cell Infect Microbiol. 2025 Oct 7;15:1667196. doi: 10.3389/fcimb.2025.1667196 (PMC12537713; doi:10.3389/fcimb.2025.1667196)
Supplement: Supplementary file 1 [file DataSheet1.docx]

**Supplementary Table S1. Information of the collected samples in this study**

| **Community** | **Sample size** | **Positive cases^a^** | **Mean ± SEM^b^**  **(years)** | **Male^c^**  **(no.)** | **Female^d^**  **(no.)** |
| --- | --- | --- | --- | --- | --- |
| Dawang | 184 | 3 | 48.1 ± 1.2 | 58 | 96 |
| Fenghua | 39 | 1 | 52.5 ± 1.5 | 11 | 27 |
| Xinghai | 22 | 0 | 46.5 ± 2.5 | 7 | 15 |

^a^, positive for isolation of tigecyline-resistant isolates;

^b^, SEM = Standard Error of the Mean;

^c^, Gender data reflect absolute counts;

^d^, Gender information was collected through voluntary self-reporting. Participants who declined to disclose their gender were recorded as 'Unknown' to comply with ethical guidelines on personal data privacy.

**Supplementary Table S2. Antimicrobial susceptibility results of *tet*(X4)-positive *Escherichia coli* isolates**

| **Strain** | **AMP (≥32)** | **CTX (≥4)** | **MEM (≥4)** | **GEN (≥8)** | **AMI (≥16)** | **STR (>16)*** | **TET (≥16)** | **TIL (>0.5)*** | **CHL (≥32)** | **NAL (≥32)** | **CIP**  **(≥1)** | **CL**  **(≥4)** | **FOS (≥512)** | **SXT (≥4)** |
| --- | --- | --- | --- | --- | --- | --- | --- | --- | --- | --- | --- | --- | --- | --- |
| SZ22HTE1 | >128 | 0.03 | 0.015 | 1 | 2 | 32 | 128 | 8 | 128 | 16 | 0.25 | 0.125 | 2 | 32 |
| SZ22HTE2 | >128 | 0.03 | 0.03 | 1 | 2 | 32 | 128 | 8 | 128 | 16 | 0.25 | 0.125 | 2 | 64 |
| SZ22HTE3 | 128 | 0.03 | 0.03 | 2 | 2 | 128 | 128 | 8 | 128 | 16 | 0.5 | 0.125 | 2 | 64 |
| SZ22HTE4 | 128 | 0.06 | 0.015 | 1 | 2 | 128 | 128 | 32 | 128 | 16 | 0.125 | 0.25 | 2 | 1 |

AMP: Ampicillin; CTX: Cefotaxime; MEM: Meropenem; GEN: Gentamicin; AMI: Amikacin; STR: Streptomycin; TET: Tetracycline; TIL: Tigecyline; CHL: Chloramphenicol; NAL: Nalidixic acid; CIP: Ciprofloxacin; CL: Colistin; FOS: Fosfomycin; SXT: Trimethoprim-sulfamethoxazole.

The numbers below the antibiotic abbreviations represent the resistance breakpoints for each drug.

* indicates that results are interpreted using EUCAST epidemiological cut-off values (streptomycin) set for *E. coli* or Clinical breakpoints (tigecycline) for *Enterobacterales*, the remaining antimicrobial agents are interpreted using CLSI breakpoints. *E. coli* ATCC 25922 was served as the quality control strain.

**Table S3. Complete genome sequences of *tet*(X4)-positive *Escherichia coli* isolates in this study**

|  | **ST** | **Size(bp)** | **Resistance genes** | **Replicons** |
| --- | --- | --- | --- | --- |
| **SZ22HTE1** | ST201 |  |  |  |
| chromosome |  | 4,741,023 | none | NT |
| pSZ22HT1-1 |  | 106,177 | *bla*_TEM-1B_, *aadA1*,*aadA2*,*tet*(X4),*tet*(A), *tet*(M), *cmlA1*, *floR*, *sul2*, *sul3*, *dfrA12*, *erm*(42) | IncY |
| pSZ22HT1-2 |  | 47,746 | *qnrS1* | IncX1 |
| pSZ22HT1-3 |  | 7,395 | none | NT |
| **SZ22HTE2** | ST877 |  |  |  |
| chromosome |  | 4,665,275 | *strAB*,*tet*(B) | NT |
| pSZ22HT2-1 |  | 190,711 | *bla*_TEM-1B_, *aadA22*, *qnrS1*, *tet*(X4), *floR*, *lnu*(G) | IncFIA8-IncHI1/ST17 |
| pSZ22HT2-2 |  | 109,111 | none | NT |
| pSZ22HT2-3 |  | 41,141 | *bla*_TEM-1B_, *aadA1*, *aadA2*, *qnrS1*, *cmlA1*, *sul3*, *dfrA12* | IncX1 |
| **SZ22HTE3** | ST10 |  |  |  |
| chromosome |  | 4,703,104 | none | NT |
| pSZ22HT3-1 |  | 201,009 | *bla*_TEM-1B_, *aadA22*, *qnrS1*, *tet*(X4), *floR*, *lnu*(G) | IncFIA8-IncHI1/ST17 |
| pSZ22HT3-2 |  | 35,337 | *aadA2*, *qnrS1*, *tet*(A), *sul3*, *dfrA12* | IncX1 |
| **SZ22HTE4** | ST1308 |  |  |  |
| chromosome |  | 4,732,882 | none | NT |
| pSZHT4-1 |  | 192,057 | *bla*_TEM-1B_, *aadA22*, *qnrS1*, *tet*(X4), *floR*, *lnu*(G) | IncFIA8-IncHI1/ST17 |
| pSZHT4-2 |  | 103,843 | none | F18:A-:B53 |
| pSZHT4-3 |  | 51,689 | *tet*(A), *tet*(M), *floR*, *erm*(42) | IncX1 |

NT, not typeable; pSZ22HT1-3 and pSZ22HT2-2 did not yield matches to known replicons using PlasmidFinder. Antibiotic resistance genes and replicons with >95% sequence homology and >60% coverage are shown.
